# P-1809. Strategies to Optimize Antibiotic Stewardship for Patients with COVID-19 Using a Systems Engineering Framework

**DOI:** 10.1093/ofid/ofae631.1972

**Published:** 2025-01-29

**Authors:** Michael Pulia, Meggie Griffin, Rebecca Schwei, Sarah Scalzo, Helena Ikenberry, Ashleen Kaur, Aurora E Pop-Vicas, Lucas Schulz, Nicole Werner

**Affiliations:** University of Wisconsin-Madison, Madison, Wisconsin; University of Wisconsin - Madison, Madison, Wisconsin; University of Wisconsin-Madison, Madison, Wisconsin; University of Wisconsin Madison, MADISON, Wisconsin; University of Wisconsin Madison, MADISON, Wisconsin; University of Wisconsin Madison, MADISON, Wisconsin; University of Wisconsin School of Medcine and Public Health, Madison, WI; University of Wisconsin Hospital and Clinics, Madison, Wisconsin; Indiana University School of Public Health-Bloomington, Bloomington, Indiana

## Abstract

**Background:**

The COVID-19 pandemic stressed antimicrobial stewardship (AMS) programs with increased workloads and lack of guidance on the treatment of COVID-19. Throughout the pandemic, COVID-19 patients have received antibiotics for this viral illness despite reported low rates of confirmed bacterial co-infections. The purpose of this study was to identify challenges to and strategies for successful antibiotic stewardship for COVID-19 inpatients.

Part 1 of Table 1

**Figure:**
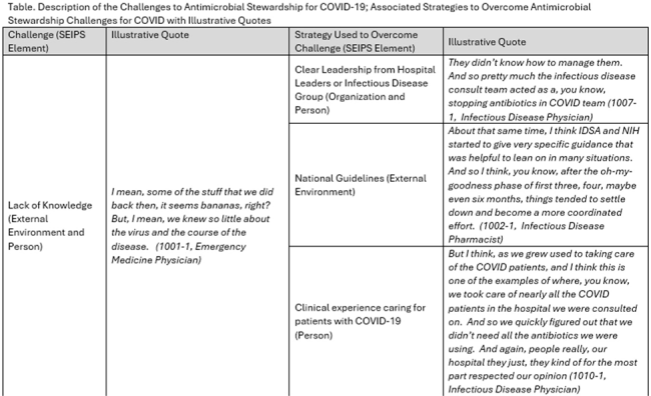

**Methods:**

We utilized the Premier Healthcare Database to identify hospitals with appropriate antibiotic prescribing before and maintained during the pandemic while facing significant COVID-19 patient burden. We conducted semi-structured interviews with pharmacists, physicians, and quality leaders involved in AMS during the pandemic. Interview guides were developed according to participants’ roles using the Systems Engineering Initiative for Patient Safety (SEIPS) framework. Interviews were recorded, transcribed, and analyzed iteratively using directed content analysis guided by the SEIPS model.

Part 2 of Table 1

**Figure:**
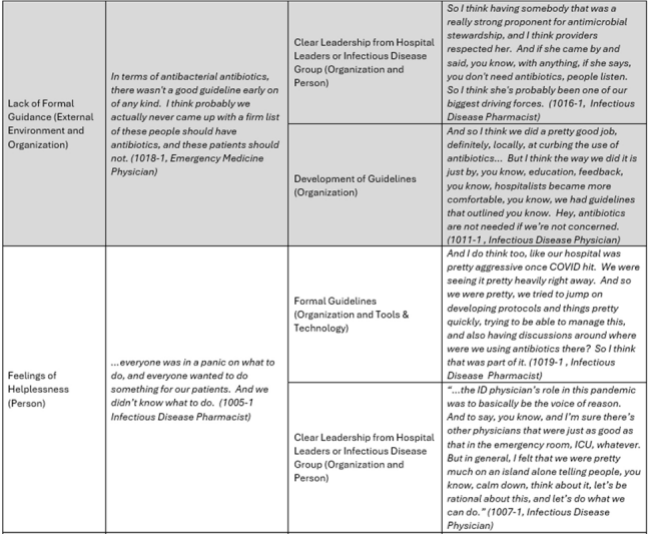

**Results:**

We conducted 30 interviews from 19 different high-performing health care systems across the country. We interviewed 17 pharmacists, 12 physicians and 1 nurse. The main challenges participants identified included lack of knowledge and formal guidance on how to treat COVID-19, feelings of helplessness, and diagnostic uncertainty. Strong, clear leadership from hospital leaders or infectious disease groups, organizational and national guidelines and clinical experience caring for COVID-19 helped providers overcome lack of knowledge, and guidelines on how to treat COVID-19 assuaged feelings of helplessness. Diagnostic tools including host response biomarkers (e.g. procalcitonin) and chest imaging were utilized and incorporated into workflows to help overcome diagnostic uncertainty and guide antibiotic prescribing.

Part 3 of Table 1

**Figure:**
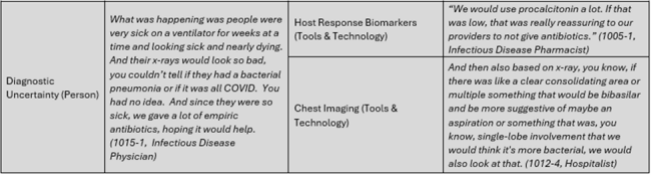

**Conclusion:**

Successful strategies for COVID-19 antibiotic stewardship included overcoming knowledge gaps with clear leadership from infectious disease teams and implementation of structured COVID-19 diagnostic and treatment guidelines.

**Disclosures:**

**All Authors**: No reported disclosures

